# Localization of sequence variations in PGC-1α influence their modifying effect in Huntington disease

**DOI:** 10.1186/1750-1326-6-1

**Published:** 2011-01-06

**Authors:** Hong Van B Che, Silke Metzger, Esteban Portal, Carolin Deyle, Olaf Riess, Huu Phuc Nguyen

**Affiliations:** 1 Department of Medical Genetics, University of Tuebingen, Calwerstr. 7, 72074 Tuebingen, Germany

## Abstract

**Background:**

Huntington disease (HD) is caused by a polyglutamine expansion of more than 35 units in the huntingtin protein. This expanded repeat length inversely correlates with the age-at-onset (AAO), however, additional genetic factors apart from the expanded CAG repeat size are thought to influence the course and the AAO in HD. Until now, among others, the gene encoding PCG-1α (*PPARGC1A*) was shown to modify the AAO in two independent, however small, populations. PGC-1α is involved in the induction of various mechanisms regulating mitochondrial biogenesis and oxidative stress defence. Furthermore, several studies have linked impairment of its function and/or its expression to HD pathogenesis. As the identification of distinct modifiers in association studies is largely dependent on the size of the observed population, we investigated nine different single nucleotide polymorphisms (SNPs) in *PPARGC1A *in order to replicate the disease modifying effect in more than 800 European HD patients and to identify an association with AAO in HD.

**Results:**

Two SNPs, one in the promoter and one in the transcribed region of the gene, showed a significant effect on the AAO. While the minor allele of SNP rs7665116 (g.38570C), located in the transcribed gene region, was associated with a delay in disease onset, especially in HD patients with Italian ancestry, the minor allele of SNP rs2970870 (g.-1437C) in the promoter region leads to an earlier onset of HD in its homozygous state. Additionally, global testing of haplotype block 2, which covers the main part of the transcribed region of the gene, revealed an association between block 2 haplotypes and the disease onset.

**Conclusion:**

Therefore, our results indicate opposing modifying influences of two SNPs within one gene on AAO and support the idea that PGC-1α dysfunction is involved in HD pathology.

## Background

Huntington disease (HD) is one of the most common autosomal-dominantly inherited neurodegenerative disorders characterized by progressive behavioural and cognitive impairments as well as motor disturbances with a relentlessly fatal course of 15 to 25 years after disease onset. The genetic defect of HD is caused by the expansion of an unstable CAG repeat in the HD gene resulting in an elongated polyglutamine tract in the huntingtin protein [1]. The onset of the disease mainly depends on the length of the CAG repeat, which is inversely correlated to the age-at-onset (AAO), and depending on the examined population, up to 73% of the variance in the AAO is determined by the CAG repeat size [2,3]. Similar to other neurodegenerative diseases, the disease causing gene alone does not fully determine the AAO and the course of the disease. The rest of the variance in the AAO, which is not accounted for by the mutant gene, is determined by other disease-modifying factors and seems to be strongly heritable [4]. To date, several genetic modifiers, particularly for the AAO of HD have been identified. They include the genes encoding the huntingtin-associated protein-1 (HAP1) [5], the adenosinergic A_2A _receptor (ADORA2A) [6,7], ubiquitin C-terminal hydrolase 1 (UCHL1) [8,9] or autophagy-related protein 7 (Atg7) [10]. The functions of the respective gene products are related to various mechanisms, which are thought to be linked to the pathogenesis of HD.

Another genetic modifier, recently identified by two independent association studies, is *PPARGC1A*: the gene encoding the peroxisome proliferator-activated receptor γ coactivator 1α (PGC-1α) [11,12]. PGC-1α, initially discovered as a key regulator of adaptive thermogenesis [13], additionally induces the transcription of cellular mechanisms regulating respiration and mitochondrial biogenesis as well as oxidative stress defence [14,15]. In HD both mitochondrial dysfunction and oxidative stress are involved in the pathogenesis of the disease [16,17]. Interestingly, PGC-1α knock-out mice display impaired mitochondrial function, striatal degeneration and a hyperkinetic movement disorder, which are features also observed in HD [18,19]. Furthermore, several studies revealed that impaired PGC-1α function and/or expression can be found in striatal cell lines, transgenic mouse models of HD and in post-mortem brain tissue from HD patients due to interference with mutant huntingtin [20-22]. Lack of PGC-1α expression even aggravates the phenotype of transgenic HD mice, whereas PGC-1α overexpression can rescue some aspects of the striatal pathology [21]. Therefore, an association between PGC-1α function and HD pathogenesis seems to be beyond controversy.

Last year, first Weydt and colleagues [12] and later Taherzadeh-Fard et al. [11] connected PGC-1α with the AAO of HD. They identified different polymorphisms to be associated with the AAO in two cohorts of HD patients (around 400 patients each). As *PPARGC1A *is located on chromosome 4p15.1-2, it is also localized near a potential modifier region [23,24]. Overall, the two modifier studies mentioned above examined a large set of single nucleotide polymorphisms (SNPs) in *PPARGC1A*, but not all the SNPs selected were included in both studies. Therefore, the aim of the present study was to replicate the association of PGC-1α with the AAO of HD in a large group of more than 800 European HD patients.

## Results

**Figure 1:**
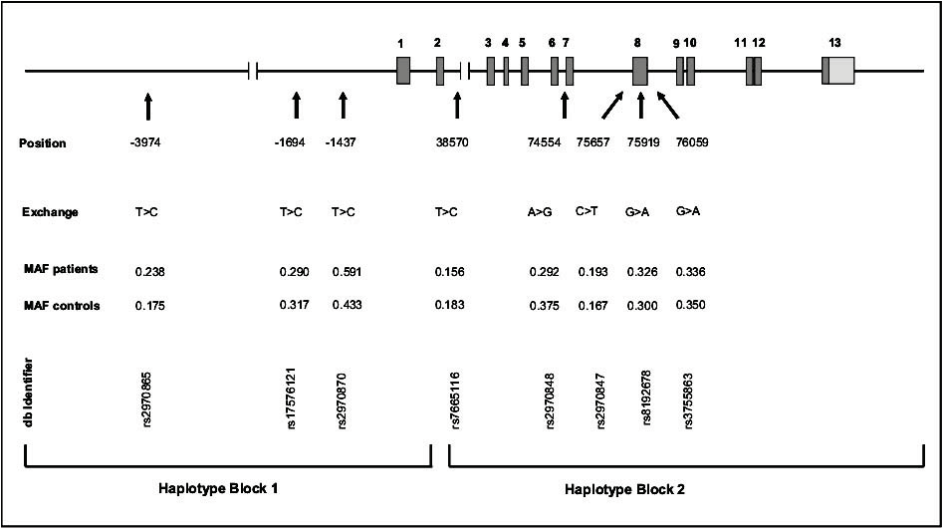
**Polymorphic sites and haplotype blocks in PPARGC1A**. Linear map with exons (full boxes) and SNP positions relative to the translational start site. Only rs8192678 results in an amino acid change (G482S). MAF indicates minor allele frequency; SNP qualifiers refer to database entries http://www.ncbi.nlm.nih.gov/SNP/. Allele and genotype frequencies of rs7665116 (P = 0.0496) and rs2970870 (P = 0.0012) in HD patients differ significantly from the respective frequencies measured in 60 healthy controls. The localisation of the two haplotype blocks is shown at the bottom. Scales differ for the transcribed sequence and the 5'-untranscribed promoter sequence.

In addition to the pathogenic CAG repeat expansion in the *HD *gene, other genetic factors play a significant role in determining AAO in HD. In our present study with a cohort of 854 European HD patients, the length of the expanded CAG repeat significantly accounts for approximately 51% of the variance in the AAO (R^2 ^= 0.5128, P ≤ 0.0001; Table 1), therefore the residual variance of disease onset has to be influenced by additional factors. In order to identify further genetic modifiers, we examined nine different SNPs in the gene encoding PGC-1α. With the exception of one SNP (rs6821591), all the others were confirmed to be polymorphic with a frequency of ≥1% in 60 healthy controls (CEPH) and were thus examined in the HD patients with regard to a possible effect on the AAO (Figure 1).

**Table 1:** Analysis of covariance of PGC1α single nucleotide polymorphisms

Model	Localization	R^2^	ΔR^2^	P value
HD CAG		0.5128		< 0.0001*
				
HD CAG + PGC-1α				
rs2970865	promoter region	0.5124	-0.0004	0.6436
rs17576121	promoter region	0.5187	0.0059	0.0795
rs2970870	promoter region	0.5180	0.0052	0.0566
**rs7665116**	intron 2			
**1-1 vs 1-2 vs 2-2**		**0.5257**	**0.0129**	0.0389*
**1-1 vs 1-2 + 2-2**		**0.5256**	**0.0128**	0.0112*
rs2970848	intron 7	0.5150	0.0022	0.1355
rs2970847	exon 8	0.5256	0.0128	0.0947
rs8192678	exon 8	0.5060	-0.0068	0.9863
rs3755863	exon 8	0.5063	-0.0065	0.0953

The level of significance was set to *P *= 0.05; *n *= 854

*HD CAG *expanded CAG allele in huntingtin

An effect of the respective PGC-1α polymorphisms on the AAO of the examined HD patients was calculated by applying an analysis of covariance together with the influence of the expanded HD allele. In this first analysis rs7665116, which is located at the 3'-end of a highly conserved region in intron 2, displayed a significant effect on the HD AAO both in an additive (P = 0.0172; Table 1) and a dominant model (P = 0.0045; Table 1). A more detailed analysis of rs7665116 revealed that its association with the AAO is primarily observed in HD patients with Italian ancestry. In this group of a total of 312 patients, rs7665116 significantly affects the onset of their first symptoms (additive model: P = 0.0172; dominant model: P = 0.0045; Table 2) and constitutes ~2.4% of the variance in the AAO. As the expanded CAG repeat in the *HD *gene explains 44.4% of the AAO in the Italian patient group (R^2 ^= 0.4444; Table 2), the SNP represents ~4.3% of the variance that can not be accounted for by the expanded CAG repeat. The minor allele g.38570C leads to a delayed disease onset of about three (T/C genotype) to five (C/C genotype) years (Table 3). Although statistically not significant, the tendency of an increase in the AAO could clearly be observed.

**Table 2:** Analysis of covariance of PGC1α rs7665116 in Italian HD patients

Model	R^2^	ΔR^2^	P value
HD CAG	0.4444		< 0.0001*
			
HD CAG + PGC1α rs7665116			
1-1 vs 1-2 vs 2-2	0.4682	0.0238	0.0172*
1-1 vs 1-2 + 2-2	0.4681	0.0237	0.0045*

The level of significance was set to *P *= 0.05; *n *= 312

*HD CAG *expanded CAG allele in huntingtin

**Table 3:** Mean ages-at-onset of the different rs7665116 genotypes in Italian HD patients

Genotype	Number of patients^a^	Mean CAGexp (SE)	Mean AAO (SE)
T/T	210	45.4 (0.33)	46.58 (0.96)
T/C	97	45.6 (0.45)	49.55 (1.43)
C/C	5	44.6 (0.98)	51.80 (2.25)

CAGexp, expanded CAG repeat number; SE, standard error; T, major allele g.38570T; C, minor allele g.38570C.

^a^n = 312.

t-test: Mean ages-at-onset do not differ significantly between the three genotypes (T/T vs T/C: P = 0.0570; T/T vs C/C: P = 0.4079; T/C vs C/C: P = 0.7596).

Apart from rs7665116 in the transcribed region of the *PGC-1α *gene, SNP rs2970870, located in the promoter region, showed a marginally significant effect on the HD AAO in the first analysis of covariance (P = 0.0566; Table 1). Under closer scrutiny, rs2970870 revealed a significant effect on the AAO of HD in patients with ≤55 CAG repeats in an additive statistical model (p = 0.0435; Table 4). As the variability of the AAO is much higher in patients with CAG repeats less than 55 units, the CAG repeat in the HD gene only determines about 38% of this variability (R^2 ^= 0.3807; Table 4). Almost 1% of the variance in the AAO of HD can be attributed to the effect of rs2970870. In the examined group, patients homozygous for the minor allele g.-1437C developed their first symptoms on average at the age of 43.7 years, which is about four years earlier than that of patients with other genotypes (Table 5).

**Table 4:** Analysis of covariance of PGC1α rs2970870 in HD patients with ≤ 55 CAGs

Model	R^2^	ΔR^2^	P value
HD CAG	0.3807		< 0.0001*
			
HD CAG + PGC1α rs2970870			
1-1 vs 1-2 vs 2-2	0.3857	0.0050	0.0435*
1-1 vs 1-2 + 2-2	0.3808	0.0001	0.7566

The level of significance was set to *P *= 0.05; *n *= 784

*HD CAG *expanded CAG allele in huntingtin

**Table 5:** Mean ages-at-onset of the different rs2970870 genotypes in HD patients with ≤ 55 CAGs

Genotype	Number of patients^a^	Mean CAGexp (SE)	Mean AAO^b ^(SE)
T/T	262	44.9 (0.21)	46.37 (0.77)
T/C	404	44.5 (0.15)	48.19 (0.62)
C/C	118	45.4 (0.33)	43.67 (1.15)

CAGexp, expanded CAG repeat number; SE, standard error; T, major allele g.-1437T; C, minor allele g.-1437C.

^a^n = 784.

^b^t-test: Patients ≤ 60 CAG repeats and genotype C/C differ significantly from patients with T/T and T/C genotype (p = 0.0039).

Several studies previously identified two haplotype blocks within the PGC-1α gene [12,25,26]. SNP rs2970870 is located in haplotype block 1, which comprises the promoter region of the gene, while rs7665116 is localized in haplotype block 2, covering the main part of the transcribed region. In our patients group, we identified a total of seven haplotypes with frequencies >0.01 in haplotype block 2. While no associations were found between haplotype block and the AAO of HD, global testing suggested an association between block 2 haplotypes and the disease onset (Table 6). Particularly, haplotype-specific statistic scores were highest and lowest for haplotypes 2-1-1-1-1 and 1-1-1-1-2, respectively. Although the choice of the examined haplotype block 2 SNPs in this study did not coincide totally with a previous study by Weydt et al. [12], the specificity and the association with HD AAO of the different haplotypes are comparable.

**Table 6:** Analysis of PGC1α Block 2 Haplotypes on HD age-at-onset

Haplotype	Frequency	Score	P value
1-1-1-1-2	0.2072	-1.892	0.0585
1-2-1-1-1	0.1550	-1.636	0.1018
1-1-1-2-1	0.1469	-1.218	0.2232
1-1-1-1-1	0.1269	0.749	0.4538
1-1-2-1-1	0.0725	1.125	0.2606
1-1-1-2-1	0.0665	1.724	0.0847
2-1-1-1-1	0.0511	2.886	0.0039

n = 729; adjusted for CAG repeat size (CAGlog)

P = 0.0113 for global haplotype statistics; block 2 haplotypes: 1, more common allele (+38570T, +74774A, +75657C, +75919G, +76059G); 2, less common allele (+38570C, +74554G, +75657T, +75919A, +76059A)

## Discussion

Genetic association studies provide the possibility to identify genetic factors that modify a disease apart from the causative gene. However, some critical aspects should be considered in association studies. A disease-associated polymorphism might have varying effects in different populations and the size of an examined patient cohort has to be large enough to detect a real association. Additionally, all studies require the replication of an identified association in another collective of patients. Until now, several studies succeeded in identifying various genetic modifiers for HD, which are involved in the different pathways that contribute to the pathogenesis of HD.

Here, we replicated the association of common variants in the *PPARGC1A *gene with the AAO of HD and can confirm a modifying effect in more than 800 European HD patients. Specifically, two polymorphisms showed a significant effect on the AAO. SNP rs7665116, located in the transcribed region of the gene, is associated with a delay in disease onset particularly in Italian HD patients and accounts for ~4.3% of the variance in the HD AAO that cannot be explained by the CAG repeat in the *HD *gene. This result is in agreement with findings reported by Weydt and colleagues [12], who also found this effect in HD patients of Italian descent. The second polymorphism rs2970870, located in the promoter region of *PPARGC1A*, modifies the AAO in patients with ≤55 CAG repeats in the mutated *HD *gene and determines almost 1% of the variance in the AAO. Due to the higher variability of AAO in HD patients with less than 55 CAG repeats and who have a disease onset in adulthood this facilitates the detection of other factors apart from the expanded CAG repeat and therefore especially small modifying effects can be more easily detected in this cohort. While we could replicate a modifying effect of the polymorphism rs7665116, the influence of rs2970870 was not described yet in previous studies [2,11,12].

Recent studies show that PGC-1α, which induces the expression of genes involved in mitochondrial energy metabolism in almost every cell type and tissue with high energy oxidative capacity, could be implicated in common disease states such as diabetes or heart failure [26-28]. Several findings also point though, to its involvement in the pathogenesis of neurodegenerative diseases such as HD. Mutant huntingtin was found to lead to an inhibition of the PGC-1α system in cell and mouse models of HD as well as in post-mortem brains of HD patients [20-22]. Although PGC-1α is expressed in the brain and is associated with neurodegeneration through mitochondrial impairment, nothing is known about its exact transcriptional regulation there. At this time, several findings lead to two hypotheses how mutant huntingtin interferes with PGC-1α function. On the one hand, mutant huntingtin may interfere with the transcription of PGC-1α reducing its expression [20,21,29]. On the other hand, mutant huntingtin may bind directly to PGC-1α impairing its ability to regulate the expression of its downstream target genes [22]. The first hypothesis is supported by the observation of Cui at al. [21] that mutant huntingtin represses CRE-mediated PGC-1α expression in striatal cells by interfering with the formation of the CREB/TAF4 complex critical for PGC-1α expression. Mutant, but not wild type huntingtin is associated with the endogenous PGC-1α promoter suggesting that mutant huntingtin disrupts the function of promoter bound transcription factors. Additionally, reduced expression of PGC-1α and its target genes was reported in muscle of HD patients [20]. In contrast, reduced expression of PGC-1α target genes, but not PGC-1α itself in brown adipose tissue argues for the second hypothesis [22]. The results of the present study support both discussed hypotheses as we identified a genetic association of the PGC-1α gene both via its promoter and the transcribed region. The effects of the two modification sites are oppositional to each other. The polymorphism rs2970870 in the promoter region leads to approximately four years earlier onset of HD. One could speculate that the disruption of a further transcriptional binding site by the polymorphism results in an earlier AAO and an intensified course of the disease. In contrast, haplotype block 2 of *PPARGC1A*, which covers the main part of the transcribed region of the gene, and polymorphism rs7665116, located in the transcribed region, also show an association with HD. Polymorphism rs7665116 even is associated with a three to five years delay in the disease onset and thus seems to antagonize a dysfunction of the protein.

Although the findings for a failure in PGC-1α expression predominate, there is also evidence to suggest an impaired functional ability of the protein itself. However, the two mechanisms for how mutant huntingtin may regulate PGC-1α activity are not mutually exclusive as PGC-1α may induce its own expression via a positive autoregulatory loop through calcium signalling components [30,31]. Additionally, the respective pathway regulating and determining the loss of PGC-1α function may depend on the tissue type, the amount of soluble and/or aggregated mutant huntingtin in the cell, and possibly the stage of disease. Even though expressed ubiquitously, the expression level of huntingtin varies in the different tissues and shows its highest level in brain [32,33]. It is therefore conceivable that the pathway influencing PGC-1α function may be dependent on the amount of mutant huntingtin in the respective tissue. This is closely connected to the general amount of soluble and aggregated huntingtin in the cell. Huntingtin not only is associated with PGC-1α, but also with a number of other transcription factors including CREB binding protein, SP1 or p53 [34-36], which are sequestered in huntingtin aggregates and thus repressing the transcription of their respective target genes. In order to understand and answer the question about the exact regulative pathway, further functional analyses of PGC-1α and its interacting and associated proteins are needed. Finally, all these pathways lead to mitochondrial dysfunction, abnormalities in energy metabolism and cell death providing a link to other neurodegenerative diseases such as Alzheimer or Parkinson disease.

In conclusion, the findings presented here support the association of PGC-1α with HD pathogenesis and thus mitochondrial impairment, especially in humans. As the exact mechanism of PGC-1α regulation and its interference with mutated huntingtin is unsolved and discussed contrarily, our observations provide an informative basis for both existing concepts. A modifier in the promoter region of *PPARGC1A*, encoding PGC-1α, may influence a failure in PGC-1α expression. A modification site in the transcribed region of the gene on the other hand may affect an impaired PGC-1α function towards target genes. As both regulative pathways seem to lead to mitochondrial dysfunction, the identification of the two modification sites in PGC-1α offers the possibility to further elucidate the role of PGC-1α in HD pathogenesis and for its future use as a therapeutic target.

## Methods

### HD patients

In total, we examined 854 unrelated European HD patients [5,10,37]. Of these, 352 patients were of German, 322 patients of Italian descent. The remaining 180 patients were from other European countries. For all patients, HD was clinically diagnosed and AAO was estimated as the age at which motor and/or cognitive symptoms first occurred. The mean AAO of HD patients was 45.9 (SD 13.4) and ranged from 5 to 85 years. CAG repeat lengths in the *HD *gene had been tested in all patients and CAG numbers have been standardized in a reference laboratory. The number of the expanded CAG repeats ranged from 39 to 90 with a median repeat number of 44. All participating individuals gave informed consent according to the Declaration of Helsinki.

### Genotyping

To detect potential modifying effects of the PGC-1α gene on AAO in HD we selected nine single nucleotide polymorphisms (SNPs), which are located in two distinct haplotype blocks [12,25,26]. Three SNPs in haplotype block 1 at gene positions -3974 T > C (rs2970865), -1694 T > C (rs17576121), and -1437 T > C (rs2970870), and six SNPs in haplotype block 2 at gene positions 38570 T > C (rs7665116), 74554 A > G (rs2970848), 75657 C > T (rs2970847), 75919 G > A (rs8192678), 76059 G > A (rs3755863), and 94581 C > T (rs6821591) were analysed in more than 800 HD patients as well as in 60 healthy controls (CEPH) (Figure 1). The single SNPs were particularly selected from dbSNP http://www.ncbi.nlm.nih.gov/snp/ and are consistent with SNPs analysed in two previous association studies by Taherzadeh-Fard et al. [11] and Weydt et al. [12]. In order to validate the achieved results in an additional HD patient cohort, our study included only those SNPs examined by both studies and those that were identified with a significant impact on the AAO of HD in at least one of the studies. Among variant sites, rs8192678 results in an amino acid change (Gly > Ser).

Genotyping of the SNPs rs2970865, rs17576121, rs2970870, rs7665116, rs2970848, rs3755863, and rs6821591 was performed by a high resolution melting-based PCR using LightCycler^® ^480 High Resolution Master (Roche Diagnostics, Mannheim) carried out on the LightCycler^® ^480 (Roche Diagnostics, Mannheim) following manufacturer's instructions. In order to facilitate differentiation of homozygous samples (homozygous for the major allele - homozygous for the rare allele) we used a pooling method described by Michael Liew et al. [38] that reveals the possibility to genotype homozygote samples by adding a known sample with a homozygous genotype to an unknown sample before amplification.

Genotyping of the SNPs rs8192678 and rs2970847 was performed by standard PCR conditions and a following restriction analysis using specific enzymes. The respective PCR products were incubated with 4U MspI (rs8192678) and 1U BsaWI (rs2970847) according to the manufacturer's instructions (New England Biolabs, Inc., Beverly, MA, USA).

Single correct typing results as well as ambiguous results were verified and controlled by sequencing with CEQ 8000 Dye Terminator Cycler Sequencing (Beckman Coulter Inc., USA) according to manufacturer's instructions.

To verify a frequency of ≥ 1% of the SNPs in healthy controls, we first analysed the respective SNPs in 60 control samples (CEPH). Having identified a polymorphism with ≥ 1% frequency, we determined the respective SNPs in the HD patients. Showing a frequency <1% in healthy controls, SNP rs6821591 was not further analysed in the patients group.

### Statistical analysis

Allele and genotype frequencies as well as Hardy-Weinberg distribution of the examined genotypes were investigated by Genepop version 4.0.10 http://genepop.curtin.edu.au/. Using the framework of linear models in an analysis of variance and covariance (JMP^® ^Version 7.0.1 SAS institute, Inc., Cory, NC, USA) we tested the modifying role of the different PGC-1α SNPs on the AAO of HD. First, we applied a model of analysis of covariance with the respective SNPs and the expanded HD allele as independent variables and the AAO as a dependent variable. The goodness-of-fit was evaluated by the proportion of variation in the AAO explained by the coefficient of determination (R^2^). We obtained the best fit of our data and a minimization of the residuals by logarithmic transformation of the AAO and the CAG repeat number in HD. To determine the effect of the polymorphisms on AAO by an analysis of variance and covariance, the effect of the expanded HD allele (HD CAG) was calculated alone, as well as with different SNPs. When the respective factors were added to the effect of the expanded HD allele (ΔR^2^), a change of R^2 ^indicated a relative improvement of the model. This method identified the percentage of the variance that was attributable to the candidate modifier loci when there was a significant P-value (P ≤ 0.05). Patients that could not be genotyped for single SNPs were excluded from statistical analyses. Differences in the AAO of HD within different genotypes of the analyzed polymorphisms were determined by a two-tailed t-test (JMP^® ^Version 7.0.1, SAS institute, Inc., Cory, NC, USA). In order to test associations between haplotypes and AAO, we used the haplo.stats software 1.4.4 http://mayoresearch.mayo.edu/mayo/research/schaid_lab/software.cfm.

## Competing interests

The authors declare that they have no competing interests.

## Authors' contributions

SM conceived and designed the study, performed the statistical analysis and coordinated and drafted the manuscript. HVBC carried out the molecular genetic analyses and was involved in writing the manuscript. EP participated in the statistical analysis of the data. CD participated in the genotyping. OR participated in the study design and coordination and reviewed the manuscript. HPN initiated the study and was involved in study design and coordination, interpretation of data and writing of the manuscript. All authors read and approved the final manuscript.
